# Should we still worry about the safety of GMO foods? Why and why not? A review

**DOI:** 10.1002/fsn3.2499

**Published:** 2021-07-27

**Authors:** Tadesse Fikre Teferra

**Affiliations:** ^1^ School of Nutrition, Food Science and Technology College of Agriculture Hawassa University Sidama Ethiopia

**Keywords:** CRISPR, feeding humanity, food security, GMO food safety, GMO regulations, population, sustainability

## Abstract

Global population is increasing at an alarming rate, posing a threat on the supplies of basic needs and services. However, population increase does not seem to be a common agendum of the global scientists and political leaders. People in the developed countries are more concerned about new technologies and their products. Pseudo‐threats related to the uncertainties of genetic engineering of crops and their outputs present on consumers are more audible and controversial than the real difficulties the world is experiencing at the moment and in the future. This review presents brief summaries of the real reasons to worry about and the uncertainties about genetically modified organisms. This article also presents the real uncertainties shared by consumers and scientists with respect to the past, present, and future of genetically engineered organisms. Developments in the field of precision genetics in the recent years and the implications on regulatory, breeding, and socio‐cultural dimensions of the global settings are included.

## Graphical abstract

This review article presents competing and contradicting human interests. On one hand, we are opposing great agricultural technologies such as genetically modified organisms (GMO) and the genetic engineering techniques. On the other hand, we are challenged with the need for feeding humanity into the future, where the global population and food production are not keeping pace of one another.

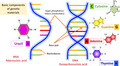

## 1 INTRODUCTION

Genetically modified organisms (GMO) have been topics of hot debates over the last few decades. Some countries have been known to have a fierce regulatory framework over the genetically modified crops. The regulations of the European Union are the ones that have been subjects of continued criticism in this regard. For instance, papers published recently argue about the basis for the EU’s regulation on the GM crops (Custers et al., 2019; Eckerstorfer et al., 2019; Halford, 2019; Hokanson, 2019; Landrum et al., 2019). It is argued that the European regulatory framework does not at present satisfy the criteria of legal certainty, nondiscrimination, and scientific adaptability (Custers et al., 2019). In 2015, the New York times carried an article with the headline: “With GMO policies, Europe turns against science” (Lynas, 2015). The European regulations do not seem to be very realistic in terms of the current challenges the world is facing in feeding the increasing global population. A predictive study conducted by the International Food Policy Research Institute indicated that by 2050, the world population reaches 9 billion and additional 70% food supply is needed than what is produced now (Ringler et al., 2010). More articles and arguments started coming out later (Hickey, 2019; Long et al., 2015; Ray et al., 2013), emphasizing the fact that the world leaders and scientists need to be worried about feeding humanity into the future and act on the use of all available technologies. This is evident that the world will not have the luxury to avoid agricultural technologies (Jacobsen et al., 2013), but need to use all available techniques without discrimination and accelerate innovation of new ones that can increase food production and productivities to be able to continue feeding humanity.

Genetically modified organisms are categories of products that came out of advanced breeding technologies, which are also categorized as precision breeding techniques (Eriksson, 2019). Traditional breeding started by simple crossing of better performing organisms with each other and stabilizing the desirable traits by self‐crossing (inbreeding), which is done several times. The first hybrid corn that was inbred several times was documented to be commercially available in the early 1920s (Anderson, 1944). Later on, breeding using mutation (alteration of genetic make ups of crops) was devised to bring about variation of performances in a population. Chemical (Ethyl methanesulfonate [EMS]), an alkylating agent that can react with cell components and cause changes to the genetics of organisms, has been in use since the 1960s (Krieg, 1963). In the mid‐20th century, ionizing electromagnetic irradiations (X‐ and gamma‐rays) were also used to cause random alteration in the genes of crops (Ulukapi & Ayse, 2015), out of which elite lines with respect to desirable traits were chosen for further breeding processes.

The science of plant genetics expanded, and the understanding of the transferability of DNA and RNA developed in the 1970s (Chassy, 2007), which later led to the development of biotechnology with a technique called “genetic engineering.” These later developments were not random alterations of genes that used to be followed by selection of elite lines and several inbreeding. The development of GMO with inserted genes from unrelated species was made possible. These later led to the development of precision genetic engineering (GE), and a very accurate specific site targeting alterations were achieved (Nakayama et al., 2014).

Today, we do not even need transferring of genes from unrelated species to bring about a desired trait in food crops or animals. The application of clustered regularly interspaced short palindromic repeat (CRISPR)‐Cas systems in genome editing has been popular since its discovery in the *Escherichia coli* genome in 1987 (Ishino et al., 2018). This review paper presents a perspective of GMO technology, associated risks, and its current status.

## 2 BASICS OF GENETIC MATERIALS

**FIGURE 1:**
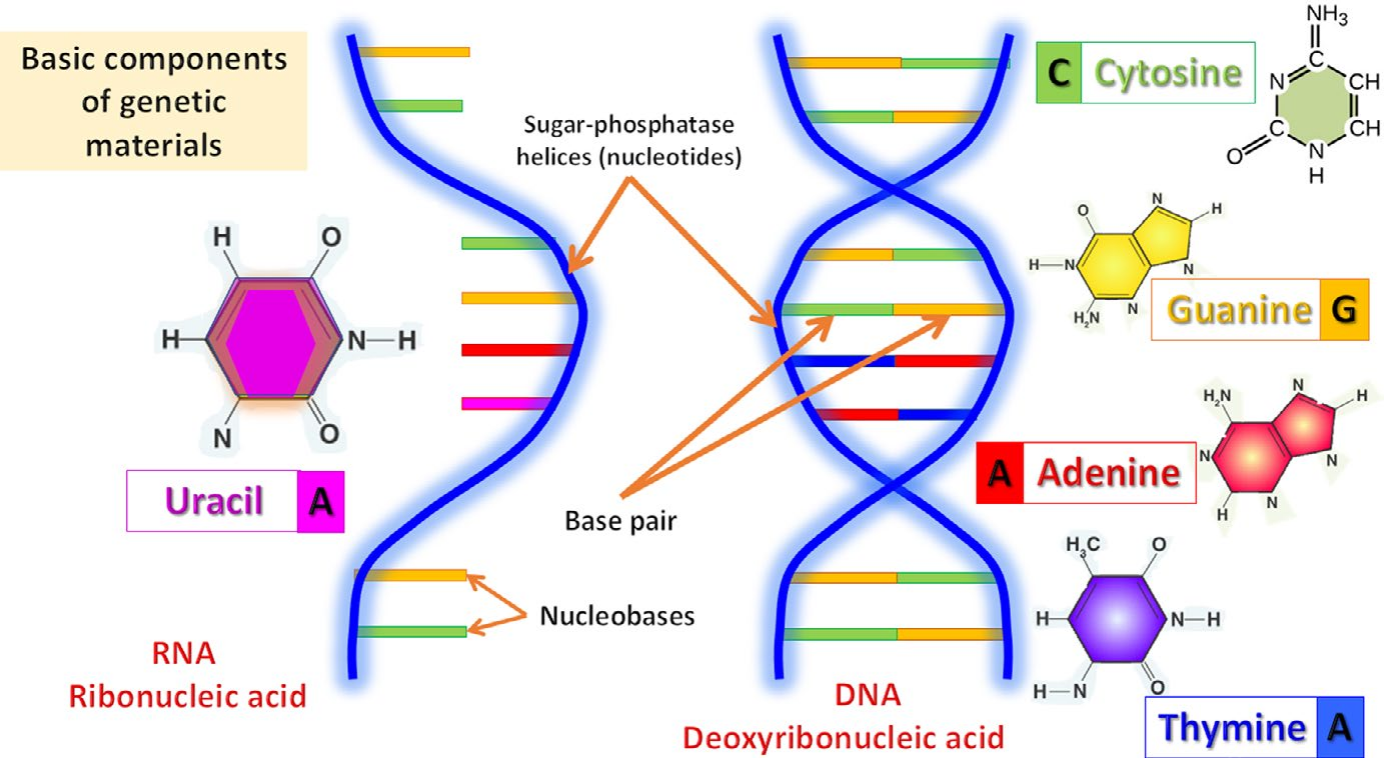
Basics of genetic materials: components and descriptions

The genetic material has basic components that collectively define the physical and biochemical properties of living entities. A gene contains a single helical stride (nucleotide) called ribonucleic acid (RNA) and a double helical nucleotide known as deoxyribonucleic acid (DNA) that are connected by a pairing bonds of four bases (cytosine [C] with guanine [G] and adenine [A] with thymine [T]) (Figure 1). The chemical bases are the building blocks of the gene, and the stretching helical nucleotides are made of pentose sugar phosphatases. The specific sequences of the bases in the gene are responsible for the formation of specific proteins that dictates the behavior of the organisms (Schjerling, 2005).

The sequences of the bases are manipulated in modern precision biotechnological techniques also known as genetic modifications (GM) or GE (Singh et al., 2006), and they naturally and randomly change through evolution (Radman et al., 2000). This review summarizes the concerns associated with the GE techniques and GMO with respect to food safety and environmental sustainability.

## 3 GENERAL REVIEW OF GM TECHNIQUES

### 3.1 Categories of GM techniques

Genetic engineering can be classified into two big categories: the transgenic and transgenic‐free types. Transgene GE involves the transfer of genetic materials from unrelated species, usually from microorganisms (bacteria and molds) associated with desirable trait into a target organism (Bock & Norris, 2018). This has been a ground breaking technology in plant breeding since the 1980s and improved agricultural production and productivity. The products of transgenic biotechnology have been termed as GMO, and the process is termed as genetic engineering (GE), GM, or biotechnology (Peter et al., 2011). This process and its products have been subjects of controversy among consumers in the developed world (Cellini et al., 2004; Eriksson, 2018; Hickey, 2019; Lynas, 2015; Van Den Eede et al., 2004; Zhao & Ho, 2005), particularly with respect to food safety. Transgenic free GM has emerged as alternative technology CRISPR/cas 9 systems, where natural or artificial genes (DNA, RNA) are used to modify genetics of the target organisms associated with desirable traits. The existence of CRISPR cas system was discovered in 1987, when an unusual repetitive DNA sequence in the *Escherichia coli* genome during an analysis of genes involved in phosphate metabolism (Ishino et al., 2018). Scientists started exploring this technology for gene editing applications only in the 2000s. The advantage of CRISPR technology is that it enables insertion and deletion of genes at much easier way than the transgenic process and also it escapes fierce regulatory procedures developed for the transgenic products.

### 3.2 Success of GM techniques—rescuing crops from invisible beasts and beyond

**FIGURE 2:**
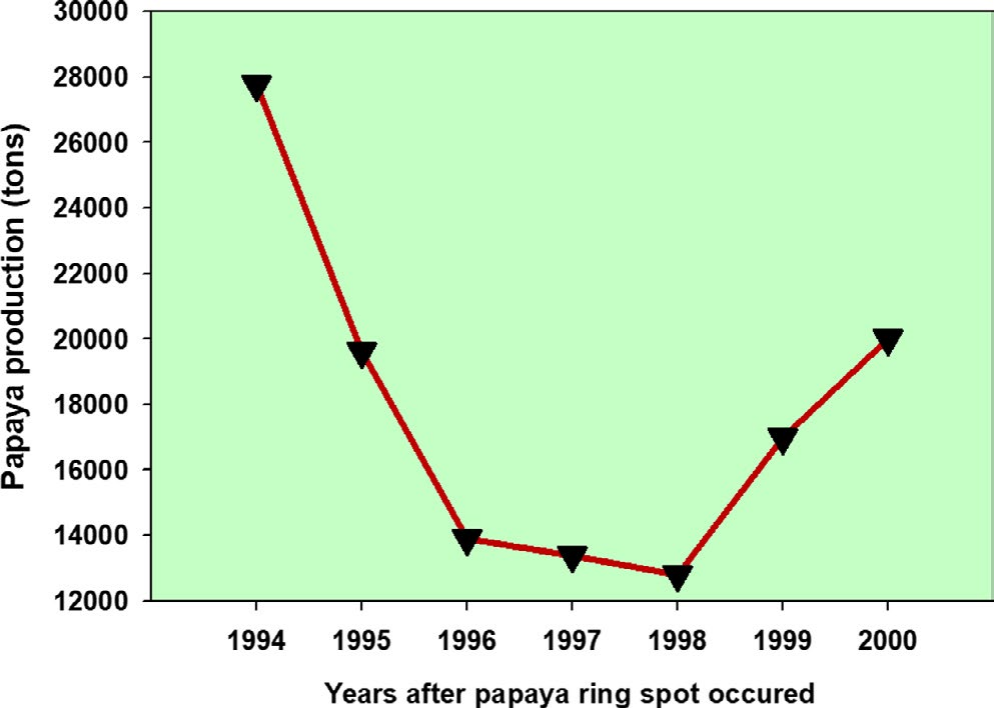
Papaya production during the ringspot disease spread

Genetic modifications has been known in agriculture for rescuing many food crops from invisible beasts that could have led to total extinction. A popular success story in this regard has been the transgenic Hawaiian Rainbow Papaya, which was developed to rescue this crop when it was devastated by ringspot virus in the 1990s (Gonsalves, 1998; Gonsalves & Ferreira, 2003). Production of papaya in Puna district of Hawaii, which was contributing 95% of the total, dropped from 27,762.5 tons in 1994 (after 2 years of the occurrence of the papaya ringspot virus, PRSV) to 12,805 tons in 1998, which was a 53.88% loss in just 4 years, Figure 2, extracted from Gonsalves and Ferreira (2003). The release of the transgenic rainbow papaya helped to revive the production to 20,000 tons, a 35.98%, increase in just 2 years.

Other success stories in rescuing plants from devastating diseases include that reported on rice against sheath blight by Liang (1998). Another potential transgenic technique for many crops (wheat, potato, carrots and tomatoes were report early on (Liang, 1998; Melchers & Stuiver, 2000)) and all these are great agricultural technologies available to the world to increase crop resistance to disease and boost production to help food production and supply to the increasing population.

The other great success of agricultural biotechnology involving transgenic crops was the biofortification of rice with beta‐carotenes (precursors of vitamin A), in eradicating preventable blindness in millions of children in developing countries (Beyer et al., 2002; Ye & Beyer, 2000). It is also clear that the principles used in rice biofortification could be applied to many more crops for the future, in efforts of feeding the world.

### 3.3 Uncertainties shared by consumers and scientists about GMOs

There are tangible uncertainties related to the science of GE and GMO products. According to Myhr (2009) and Nielsen and Myhr (2007), the types of uncertainties surrounding GE and GMOs can be divided into three broad classes:

- Reducible uncertainty, due to lack of knowledge and the novelty of the activity, which can be addressed with more research and focused collection of empirical data.
- Irreducible uncertainty due to inherent randomness, variability, and complexity in the nature of biological system under consideration.
- Uncertainty arising from ignorance given that the prevailing operating paradigms and models do not adequately represent the biological system evaluated.

However, since the start of wide exercise of modern biotechnology in the early 1980s for genetic improvement of food crops (Chassy, 2007), there have never been any direct safety hazard reported from any GE or GMOs. Moreover, governments have established the most strict testing measures for the safety of GMOs over the last decades to make sure public safety and environmental sustainability, as summarized in multiple scientific documentations (Davison & Ammann, 2017; Hartung & Schaub, 2018; Smyth & Phillips, 2014). However, the pseudo concerns over the safety and environmental sustainability of GMOs were extremely heightened by consumers and social media activism together with misconceptions aired by mainstream medias in the western world and by some governments, particularly in the European Union, which were also reported in scientific publications (Ammann, 2014; Kuntz, 2012; Masip et al., 2013; Tagliabue, 2015). Due to the strict regulations and associated hurdles created by series of tests and examinations by regulatory bodies, the GE techniques became too expensive and the time required to generate technology has been elongated. This also increased the cost of doing innovation in GE. This moved the research and development (R&D) activities in biotechnology from the public research sectors exclusively to the private corporates. Today, GE does not seem to be a technique of choice even in the corporate R&D plans, as CRISPR cas‐9 is getting popularity.

## 4 SHOULD WE STILL WORRY ABOUT GMOS? WHY AND WHY NOT?

### 4.1 Why should we still worry about GMOs?

**FIGURE 3:**
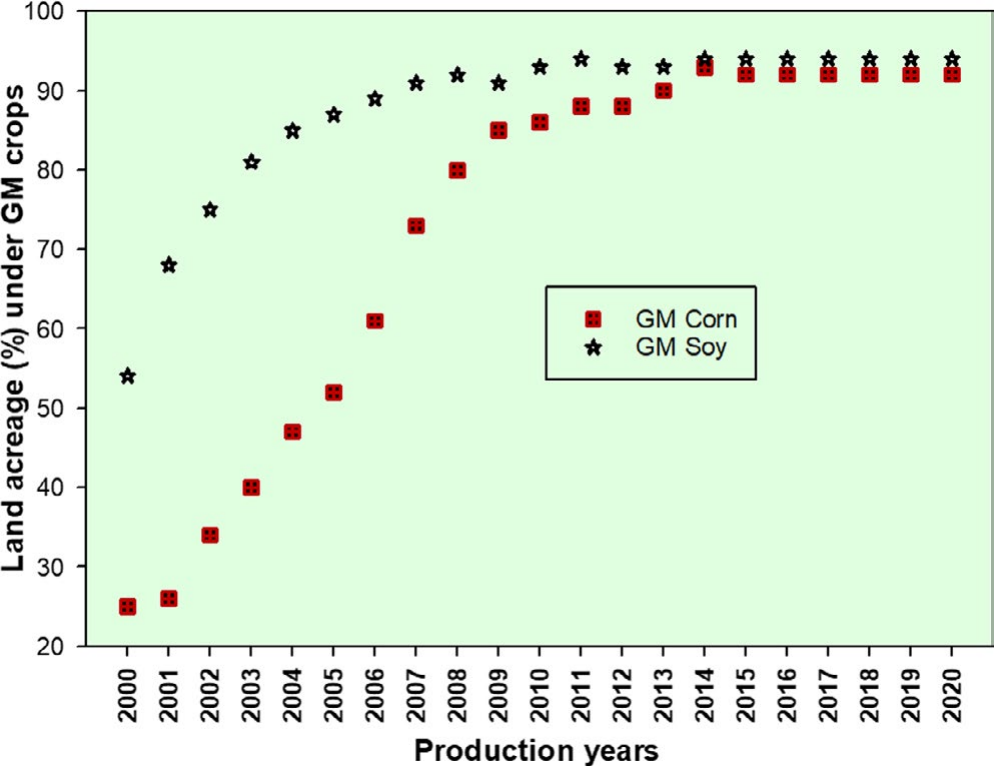
Land acreage under GM corn and soybean in the United States

If GMO crops and animals are presenting any concern to the consumer safety and/or environmental sustainability, there is no escape as GM entities are already in the environment, extensively crossing with the land races of the genetically engineered crops and their wild relatives, particularly for the cross‐pollinating crops (Castro Galvan et al., 2019; Halfhill et al., 2003; Jhala et al., 2021; Stewart et al., 2003; Wisniewski et al., 2002). The myths and realities associated with the GE of maize and more were reported by Parrott (2010). In certain countries like the United States of America, the GM crops are already massively in the production systems. The US average percent acreage under GM corn and soy bean by 2020 were reported to be 91.47 and 93.81, respectively, as documented by FDA (2020; USDA, 2020). In the FDA data, the proportion of GM corn and soy has shown steep increase between 2000 and 2013 and remained almost constant after 2014 (Figure 3).

The large proportion of GM in the crop production systems in the United States is also affecting the market destinations of corn and soy beans, the major one being Mexico. The development that the Mexican government is due to ban import of GM corn by 2024 has been a shocking news to the US market (Polansek, 2021). Corn in Mexico has already been under hot debates pertaining to the introgression of transgenic lines into the local landraces posing threat to the national corn biodiversity (Duncan et al., 2019; Mercer & Wainwright, 2008; Ortiz‐Garcia et al., 2005; Quist & Chapela, 2001). This heightens the uncertainties and concerns of GM technologies on food safety and environmental sustainability. The implication is that, if the risks of GMOs on consumer safety and natural biodiversity is real, the world has to just face it and find a way out, as there is no easy escape as of now. Rather than fragmented approaches by nations like Mexico, a global consensus is needed to support basic researches focusing on generating robust empirical data and accumulating knowledge that would potentially help in developing lasting solutions.

### 4.2 Why should we not worry about GMO?

There are several points that reduces our worries about GMOs. As discussed in the previous sections, GMO is no longer the method of choice in improving crops for better economic and technological outcomes. GE is an extremely expensive technique in terms of technologies, fierce regulations, and time requirements. There are also easily acceptable and more accurate technologies taking over the transgenic GE with no regulations required (at least for now). Since its introduction in the 1980s (Ishino et al., 2018), CRISPR cas‐9 is getting popularity as a safer and cheaper GE technique that avoids the need for transferring genetic materials from unrelated species with a lot of uncertainties. Transgenic techniques of crop improvement are getting a smoother exit pushed by multiple factors including the cost, regulations, time requirements, consumer rejections, and uncertainties associated to its products emanating from lack of complete understanding and confidence for future predictions.

## 5 WHAT IS NEW IN THE FIELD OF BIOTECHNOLOGY?

The field of molecular genetics has ever been growing and resulted into the development of new tools that enabled scientists to advance GE applications. For instance, the once difficult GE of ornamental plants was made simple in the next‐generation genome sequencing (Smulders & Arens, 2018). Details of molecular plant breeding strategies and tools are compiled into a book (Al‐Khayri et al., 2016) for more insights. The possibility of engineering crops to enhance metabolic pathways that improve human nutrition and health has been recently documented (Birchfield & McIntosh, 2020; Tatsis & O’Connor, 2016; Zheng et al., 2020). The developments in the CRISPR cas‐9 techniques in plant breeding is presenting the options of insertion and/or deletion of multiple genes at a time that are responsible for different traits (Kim et al., 2017; Shin et al., 2017). In improving wheat to eliminate gluten reactions, deletion of up to 35 different genes out of 45, identified to be responsible for gliadin synthesis (major gluten component responsible for celiac disease and wheat allergy), was possible, while immunoreactivity was reduced by 85% (Sánchez‐León et al., 2018). More detailed reports on the future prospects of CRISPR cas‐9 techniques were recently presented by Nidhi et al. (2021). More accurate applications of the new GE techniques, including CRISPR, are expected to better enhance the nutrition and health of people in the years to come.

## 6 WHAT SHOULD BE MORE CONCERNING THAN GMO?

**FIGURE 4:**
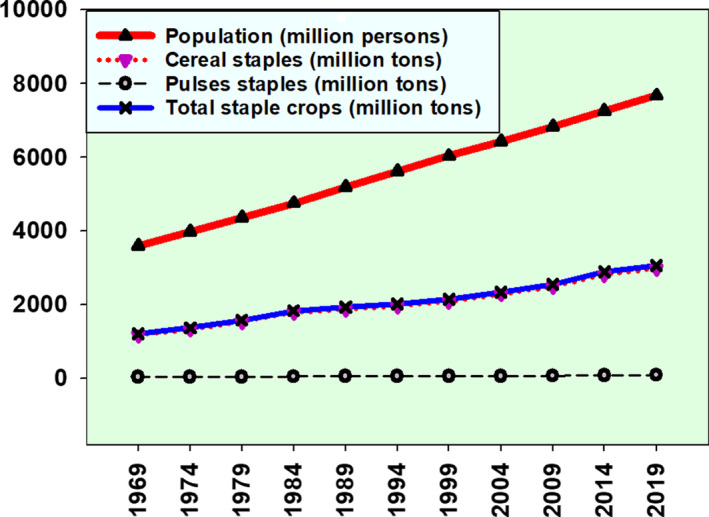
Population versus crop production, not keeping pace of each other

It is expected that the population of the globe will be reaching 9 billion in a matter of three decades. The population pressure will be more concerning to the developing world as food supply will be extremely challenging (Jacobsen et al., 2013). In those situations, the world leaders and scientists will not have the luxury of choosing agricultural technologies, but try all that are available and create new ones to increase food production. According to the population data extracted from the World Bank (WB, 2021), and staple crops production data extracted from FAOSTAT (FAO, 2021), the major staple crops (cereals and pulses) production has not been keeping pace with the global population growth over the last half century (Figure 4), regardless of the ground breaking innovations in agriculture. This implies that we need to be much more concerned about being able to continue feeding humanity into the future than choosing and accusing technologies made available to increase food production and sustain supplies.

The Europeans have been against GMO and other agricultural technologies by setting controversial regulations. The Europeans have also been promoting and funding organic and conventional agricultural practices in developing world and restricting them from producing enough toward food security (Popescu, 2019; Willer & Lernoud, 2019). Europe is the major region of the world being continuously challenged by migrations of people from developing countries and should work to support these populations toward ensuring food security, rather than dealing with migration crisis (Mavroudi & Nagel, 2016).

Ray et al. (2013) reported that yields in maize, rice, wheat, and soybean—that comprises nearly two‐thirds of global agricultural calories, are increasing at 1.6%, 1.0%, 0.9%, and 1.3% per year noncompounding rates, respectively, which is less than the 2.4% per year rate required to double global food production by 2050. Europe does not seem to be caring about the grand global challenge ahead of us, but the unrealistic “food safety” concerns associated with the GMO products.

It should be clear that the world prioritizes boosting agricultural production to be able to feed humanity, and no exception for Europe and other powers. Feeding the increasing population should be a matter of grave concern to the scientists, leaders, and the general public. It is projected that the current pace of food production and yield is not being able to keep up with the population growth (Figure 4), which is expected to hit 10 billion over the next three decades (Hickey, 2019). In addition to decisions to use all available technologies, it is a necessity that efforts are made to develop new agricultural technologies and increase food productions and yield to supply enough foods to the increasing population. In this respect, GMO technology is not just a matter of choice, but the technology with great potential to be explored towards achieving global food and nutrition security, as there are no other promising resources and mechanisms more important in achieving goal of feeding 10+ billion people around the globe, in just three decades.

## 7 SUMMARY

The hugely controversial concerns over the GMO foods in terms of consumer safety and environmental sustainability seem to remain unchanged. There are tangible reasons for the world to still worry about GMO, although new techniques emerged and are getting popularity in Biotechnology. There are also arguments that advanced GE technologies remain alternative means for increasing food production and should get the necessary attentions by the scientists and leaders at global level. Even if the transgenic GMOs are seemingly giving ways to the CRISPR edited nontransgenic GMOs that are exempted from the strict regulations, the world will remain threatened by the heavy presence of the transgenic GMOs and their potential risks. It seems that rather than worrying about the GMO food safety and environmental sustainability, the world should be worried by the increasing global population that is expected to exceed 9 billion by 2050, leaving the world short of food supply by over 70%. The population pressure, coupled with corrupt leadership in developing countries, is more concerning to sustain humanity. Worrying only about the issues of the populations in the developed world and ignoring those of the developing countries will make the world pay steeper and real prices than just a worry about uncertainties in a particular technology. Developed countries have already started dealing with immigration crises by people escaping the corrupted leaderships in Africa, Asia, and the rest of the continents. Increasing food production and health services with all the available technologies including GE should be the way forward.

## CONFLICT OF INTEREST

The author declare that he does not have any conflict of interest.

## AUTHOR CONTRIBUTION

**Tadesse Fikre Teferra:** Conceptualization (lead); Data curation (lead); Investigation (lead); Writing‐original draft (lead); Writing‐review & editing (lead).

## ETHICAL REVIEW

This study did not involve any human or animal testing.

## INFORMED CONSENT

This review article did not involve study participants, and informed consent was not required.
